# Personalized Embryo Transfer Outcomes in Recurrent Implantation Failure Patients Following Endometrial Receptivity Array With Pre-Implantation Genetic Testing

**DOI:** 10.7759/cureus.26248

**Published:** 2022-06-23

**Authors:** Jayesh Amin, Ripal Patel, Grishma JayeshAmin, Jayaprakash Gomedhikam, Swarnalatha Surakala, Muralikrishna Kota

**Affiliations:** 1 Department of Obstetrics and Gynecology, Wings IVF Women's Hospital, Ahmadabad, IND; 2 Department of Embryology, Wings IVF Women's Hospital, Ahmadabad, IND; 3 Department of Embryology, Life Fertility and Research Centre, Visakhapatnam, IND

**Keywords:** window of implantation, pre-implantation genetic testing, personalized embryo transfer, recurrent implantation failure, endometrial receptivity array

## Abstract

Introduction

Implantation failure is a trending problem for pregnancy outcomes. Women's reproduction rates can increase by in-vitro fertilization, which comes with frequent implantation failures. These failures can be mitigated by the personalization of embryo transfer depending on the patient's implantation window. The study aimed to assess the importance of using an endometrial receptivity array (ERA) combined with pre-implantation genetic testing in patients with recurrent implantation failure (RIF) and the significant role of personalized embryo transfer (PET) after ERA in patients with a displaced window of implantation. The study also determined the efficacy of this approach in improving clinical outcomes.

Methods

We conducted this observational retrospective study following approval by the Ethics Committee of Wings In-Vitro Fertilization (IVF) Women's Hospital, a unit of Reveba Infertility Clinics Pvt. Ltd., Ahmadabad (Approval No. 2019/002/31B). Two hundred ninety-one RIF patients were recruited and categorized into Group I (patients without ERA group) and Group II (ERA study group). Patients in the ERA study group were screened for ERA and subclassified into receptive and nonreceptive ERA groups. PET was performed for all subjects in the ERA study group according to their receptivity as assessed by ERA. We also screened some of the patients for ploidy (genetic) status of embryos by pre-implantation genetic testing for aneuploidy (PGT-A) before embryo transfer. The study had a power of 95% and an alpha of 0.05; therefore, 80 ± 2 subjects were required to conduct the study.

Results

The primary outcome was the clinical pregnancy rate followed by the implantation rate. We found an improved clinical pregnancy rate and implantation rate (73.5% and 78.6%) in the nonreceptive endometrial group after adjusting their embryo transfer schedule to their endometrial receptivity. The clinical pregnancy rate (64% and 65%) and implantation rate (65% and 74%) in receptive and nonreceptive ERA (respectively) were high in subjects with donor oocytes for IVF/intracytoplasmic sperm injection. In addition, patients who opted for PGT-A to eliminate the risk of transferring aneuploidy embryos had significantly better implantation (88% and 95% receptive and nonreceptive, respectively) and clinical pregnancy rates (100% in both groups) compared to non-PGT-A screened patients (p<0.05; 34% and 37% clinical pregnancy rate, 96% and 57% implantation rate in receptive and nonreceptive groups, respectively).

Conclusion

Endometrial receptivity assessment is a highly beneficial method to assess the genetic expression of the endometrium and embryo transfer timing. In our study, in patients with recurrent implantation failure, this technology found receptivity issues and provided a chance to plan embryo transfer according to the window of implantation. The combination of PGT-A with ERA rules out the genetic issues related to embryos. In RIF patients, ERA results-guided PET improved the implantation rate and reproductive outcomes.

## Introduction

In humans, implantation is the stage of reproduction at which healthy embryos adhere to the wall of the receptive endometrium. The basic and frequent reason for implantation failure is aneuploidy embryos that lack developmental competency [1]. The implantation failure of euploid embryos determines a non-embryonic source with endometrial receptivity problems, representing another crucial cause of failure [1]. The window of implantation (WOI) is not universal for all women-it varies by individual. Studies observed displaced WOI in one of four recurrent implantation failures (RIFs) [2,3]. In assisted reproductive technology (ART) cycles, embryo transfer is synchronized with the WOI. The prognosis of the cycle should be monitored carefully from clinical and laboratory aspects.

In some cases, even though the patient has good endometrium and good-quality embryos, implantation is difficult to achieve. In such cases, two major issues must be assessed. The first is embryo ploidy status by pre-implantation genetic testing for aneuploidy (PGT-A), and the second is endometrial receptivity by endometrial receptivity array (ERA). PGT-A detects whole chromosome aneuploidy and potentially increases implantation and live birth rates, and decreases the rate of early pregnancy failure [4]. RIF is a significant indication for ERA, even after transferring euploid embryos. The determination of the WOI will improve implantation success in a subsequent cycle with personalized embryo transfer (PET). PET is performing an embryo transfer when the endometrium is in the optimal receptive stage [5]. This study aimed to assess the role of ERA and any beneficial effects of PET in improving clinical pregnancy rates (CPR) and implantation rates (IR) in RIF patients. The study also aimed to delineate the CPRs between receptive and nonreceptive ERA combined with PGT analysis in RIF patients.

## Materials and methods

Study design, location, and setup

**Figure 1:**
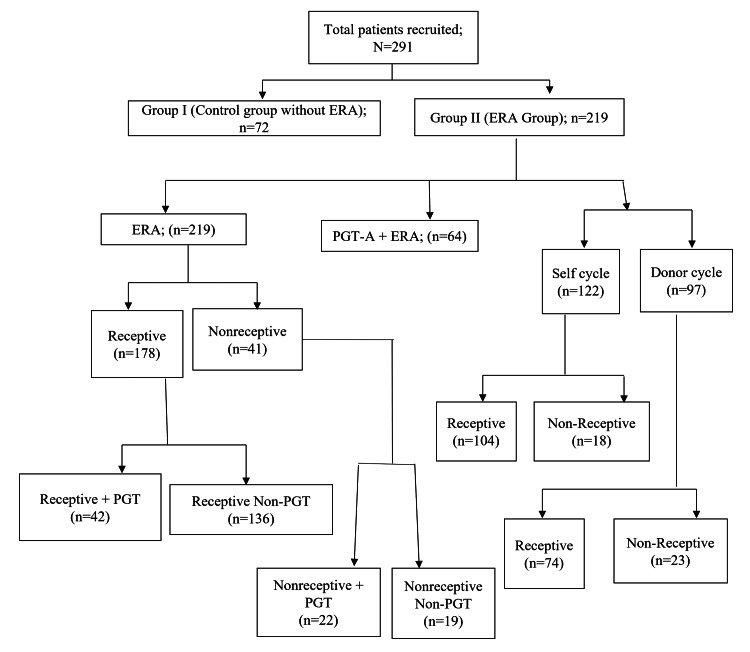
Study design flowchart ERA: endometrial receptivity array; PGT-A: pre-implantation genetic testing for aneuploidy

We conducted this observational retrospective study of RIF patients who underwent ERA from December 2019 to August 2021 at the Wings In-Vitro Fertilization (IVF) Women's Hospital, a unit of Reveba Infertility Clinics Pvt. Ltd., Ahmadabad. We retrieved patient data retrospectively from the clinic's MediTEX software (MediTEX IVF, Regensburg, Germany). The study included patients with more than two unsuccessful fresh/frozen embryo transfers with two morphologically/PGT-A normal embryos. We included both self and donor cycles. The study excluded patients with confounding factors such as hydrosalpinx, endometrial polyp, submucous fibroid, immunological disorders, previous difficult embryo transfer, and thin endometrial thickness (i.e., < 6 mm) after hormone replacement therapy. Figure 1 presents the study design flowchart.

Participants were categorized into two groups: Group I consisted of RIF patients without ERA (n=72, control group), and Group II consisted of RIF patients with ERA (n=219; Study ERA groups). The study's primary outcome measures were IR, CPR, and abortion rate (AR). The clinical outcomes of the control group (without ERA) and ERA study group and subgroup's data were compared and statistically analyzed. The study was approved by the Ethical Committee of Wings IVF Women's Hospital (Approval No. 2019/002/31B).

ERA protocol

All patients in the ERA study group started the ERA protocol on day two or day three of their menstrual cycle. Patients were advised to take one estradiol valerate tablet (2 mg) three times daily for four days, followed by twice daily for eight days. On the twelfth day of this regimen, the patient was called for endometrial assessment, hysteroscopy, and ERA. We added progesterone injections (100 mg, six injections total) on the 13^th^ day of the regimen (considered as day zero). ERA was performed on the last day/(P+5) of injection. After five days of progesterone injections, the first endometrial biopsy was collected from the uterus fundus and assessed for ERA transcriptomic analysis. According to Ruiz-Alonso et al., the ERA “consists of a customized array containing 238 genes expressed at the different stages of the endometrial cycle and is coupled to a computational predictor that can identify the receptivity status of an endometrial sample and diagnose the personalized WOI […] of a given patient regardless of the sample's histological appearance” [3,6]. The endometrial gene expression profile was processed, and the ERA computational predictor diagnosed endometrial receptivity. The ERA test results were categorized into receptive ERA and nonreceptive ERA, conveyed in terms of hours based on the day of progesterone administration (i.e., 80 to 90 hrs [± 3 hrs], 90 to 110 hrs [± 3hrs], 110 to 130 hrs [± 3 hrs], 140 to 150 hrs [± 3hrs], and 150 to 160 hrs [± 3 hrs] as P+ 3, P+4, P+5, P+6, and P+6 transfer, respectively).

Pre-implantation genetic testing and embryo screening

At Wings IVF Women's Hospital, Ahmadabad, all embryo biopsy samples were processed and assessed by next-generation sequencing (NGS) at a single standard laboratory (Igenomix, New Delhi). PGT-A determined the status of the embryo into euploidy, aneuploidy, and mosaicism. The VeriSeq NGS (Illumina, San Diego, CA) platform was used to analyze the NGS result to detect abnormal chromosome segments as small as 1.5 million base pairs. According to the biopsy, NGS screening, and analysis (and according to copy variations), euploid embryos were a chromosome with two copies, monosomy is a chromosome with one copy, and trisomy is a chromosome with three copies. Any value between one and two or two and three was classified as a mosaic. More than 80% of mosaicism is categorized as aneuploidy, <20% is euploid, and 20% to 80% are classified as a mosaic. After ERA testing, PET was done, and the clinical pregnancy of respective patients was confirmed by assessment of β-subunit of human chorionic gonadotropin level after 15 days of embryo transfer.

Statistical analysis

To analyze the data, IBM Corp. Released 2011. IBM SPSS Statistics for Windows, Version 20.0. Armonk, NY: IBM Corp. was used. The number of participants should be at least 40 (80 in total) to assure 95% of the power of the study as the lower boundary of 95% one-sided confidence interval between the differences in the combination of Receptive ERA and PGT and PGT alone groups in the parameters of clinical pregnancy rate and implantation rate. Pregnancy outcomes, including IR and CPR, were statistically compared between receptive and nonreceptive patient groups and PGT-A and without PGT-A testing using chi-squared analysis. We considered p≤0.05 as statistically significant. We drew statistical interpretations in comparison by multivariate analysis between Group I and Group II and determined their significance.

## Results

Demographics of the study group

Two hundred ninety-one patients were included in the study (Table 1). Group I (the control group) contained 72 participants with a mean age of 35 ± 4.5 years, mean body mass index of 26.4 ± 1.3 kg/m2, and mean active marriage life of 5 ± 1.6 years. The control group and Group II subjects had a mean implantation failure of 2.2 ± 0.9 and 2.4 ± 0.8, respectively.

**Table 1:** Study population demographics BMI: body mass index; ERA: endometrial receptivity array; SD: standard deviation.

Patient Demographics	Group I/Control Group; n=72 (Mean ± SD)	Group II/ERA Group; n=219 (Mean ± SD)
Average female age (years)	35 ± 4.5	36 ± 4.2
BMI	26.4 ± 1.3	27.5 ± 1.1
Active marriage life (years)	5 ± 1.6	5 ± 1.2
Average no. of failures	2.2 ± 0.9	2.4 ± 0.8

Reproductive outcomes of Group I and Group II

Patients in Group I reported a CPR of 23% (n=17), an IR of 4.1% (n=3), and an AR of 17% (n=13). Patients in Group II had a CPR of 48.4% (n=106); an IR of 50.6% (n=111), and an AR of 2.2% (n=5). Among 219 patients, 64 (29.2%) opted for a PGT-A with ERA to check the ploidy status of the embryo. For the remaining 155 patients (70.7%), a morphological selection was done to select the best embryos and planned PET based on the WOI as per the ERA results. Reproductive outcomes were significantly better in Group II patients than in Group I patients (p=0.05; Table 2).

**Table 2:** Reproductive outcomes comparisons PGT: pre-implantation genetic testing; ERA: endometrial receptivity array

Reproductive Outcomes	Group I/Control Group; n=72	Group II/ERA Group; n=219
No. of morphologically screened transferred embryos	72 (100%)	155 (70.7%)
No. of PGT transferred embryos	0	64 (29.3%)
Overall clinical pregnancy rate	17 (23%)	106 (48.4%)
Overall implantation rate	3 (4.1%)	111 (50.6%)
Overall abortion rate	13 (17%)	5 (2.2%)
p-value	0.83	0.05

CPR and IRs in receptive and nonreceptive ERA groups

Among 219 patients assessed for ERA, 178 were receptive, and 41 were nonreceptive as per ERA results. Of the 178 receptive ERA patients, 83 reported positive pregnancy 15 to 18 days after embryo transfer, and all had confirmed implantation (47%). However, in the nonreceptive ERA group, patients were regrouped under three headings according to their PET timing. The only patient with P+3 turned positive and showed successful implantation. The second group was P+4 (n=27), where 18 (66.6%) had implantation. The third group was P+6 (n=13), where nine (69.2%) had implantation. For all ERA nonreceptive patients, the total IR was 28 of 41 (68%; Table 3), higher than the ERA receptive group but not significantly higher. This trend is the same, with a CPR of 45.5% compared to 60.9% in the ERA receptive and nonreceptive patients. We found no significant difference in AR between the ERA receptive and nonreceptive groups (Table 3).

**Table 3:** Clinical outcomes in ERA receptive and ERA nonreceptive patients ERA: endometrial receptivity array; PET: personalized embryo transfer; IR: implantation rate; CPR: clinical pregnancy rate; AR: abortion rate.

Group	PET	IR, n (%)	CPR, n (%)	AR, n (%)	Negative Pregnancy, n (%)
Receptive (n=178)	P+5 (n=178)	83 (47%)	81 (45.5%)	2 (2.4%)	95 (53.3%)
Nonreceptive (n=41)	P+3 (n=1)	1 (100%)	1 (100%)	0 (0%)	0 (0%)
	P+4 (n=27)	18 (66.6%)	16 (59.2%)	2 (11.1%)	9 (33.3%)
	P+6 (n=13)	9 (69.2%)	8 (61.5%)	1 (11.1%)	4 (30.7%)

ERA outcome in donor and self-oocyte cycles

We also analyzed data by respective type of gametes (self-gametes, n=122; egg donor cycles, n=97) transferred into the patient and their respective IRs. The IRs are high in the donor gametes group (64.8% in the ERA receptive group, 73.9% in the ERA nonreceptive group) compared to the self-gametes (33.6% in the ERA receptive, 61.1% in the ERA nonreceptive group) regardless of endometrial receptivity (Table 4). Likewise, the CPRs were 64% in the donor group and 33% in the self-gamete groups among the ERA receptive participants and 65% in the donor versus 56% in the self-gamete groups in the ERA nonreceptive participants.

**Table 4:** Clinical outcomes between self and donor cycles in ERA receptive and ERA nonreceptive patients ERA: endometrial receptivity array; IR: implantation rate; CPR: clinical pregnancy rate; AR: abortion rate

Study Group	IR, n (%)	CPR, n (%)	AR, n (%)
Receptive donor (n=74)	48 (65%)	47(64%)	1 (1.3%)
Receptive self (n=104)	35 (34%)	34 (33%)	1 (0.9%)
Nonreceptive donor (n=23)	17 (74%)	15 (65%)	1 (4.3%)
Nonreceptive self (n=18)	11 (61%)	10 (56%)	2 (11.1%)

CPRs in patients of ERA combined with PGT-A

Prenatal genetic screening was advocated for all the subjects; however, a minority of patients (29.2%) opted for PGT-A, whereas most (70.7%) opted for embryo selection based on the morphological criterion of embryos for transfer (Table 1). Interestingly, embryos screened by PGT-A and optimally transferred into the recipient resulted in high IR in both the groups, regardless of endometrial receptivity. The primary clinical outcomes, including IR and CPR for receptivity and nonreceptivity with PGT-A, were more significant and showed better results in positive pregnancy rates than without PGT-A (p≤0.05; Table 5).

**Table 5:** Pregnancy outcome in PGT-A patients and Non-PGT-A patients IR: implantation rate; CPR: clinical pregnancy rate; PGT-A: pre-implantation genetic testing for aneuploidy

Content	Receptive + PGT-A (n=42)	Receptive –PGT-A (n=136)	P-value	Nonreceptive +PGT-A (n=22)	Nonreceptive - PGT-A (n=19)	P-value
CPR	37 (88%)	46 (34%)	0.008	21 (95%)	7 (37%)	0.08
IR	37 (88%)	44 (96%)	0.005	21 (100%)	4 (57%)	0.01

## Discussion

Studies on the evaluation of endometrial receptivity by genomic analysis in RIF patients of the Indian subcontinent are scarce. The overall IR observed in our study was 50.6%, slightly lower than that reported by Tan et al. (63.3%) [7] but higher than reports by Patel et al. (39%) [5] and Ruiz et al. (33.9%) [3]. RIF is an implantation failure of good-quality embryos in a minimum of three reproductive treatment cycles [8]. Various elements affect implantation, including maternal, paternal, and embryonic factors [9]. The primary components of concern in RIF are embryo potential and endometrium receptivity. Previous studies documented the efficiency of the ERA in assessing the WOI, and better adjustment of embryo transfer timing improved IR [10]. The assessment of embryo ploidy status allowed for improved assessment of endometrial contribution for RIF. NGS-based embryo PGT-A represents a valuable supplement to the current management of RIF [11]. Our study assessed endometrial receptivity and embryo ploidy status in RIF patients by ERA and PGT-A, respectively, and assessed their reliability. According to the ERA assessment in our cohort, 18.7% of patients have displaced WOI (nonreceptive), which is comparable to that reported by Ruiz et al. (25.9%) [3]. We found a high rate of implantation and continuation of pregnancy in the nonreceptive endometrium group after adjusting their embryo transfer to PET as per their WOI.

The primary outcome of the study is to determine whether PGT or ERA plays a significant role to improve clinical outcomes of IVF in repeated implantation failure patients. In the Indian Scenario, the vital cause of RIF is socio-economic status, late marriage, increased environmental pollution, and improper nutrition at the early stages of the growth phase, which leads to miscarriage and abortions. Therefore, present tests available for IVF patients advised by clinicians are ERA and PGT to analyse their uterine receptivity and genetic abnormalities. The evaluation of genetic testing is useful to select the good embryos for embryo transfer and successful pregnancy rates. ERA findings help to assess the uterine abnormality and receptivity nature by identifying the window of implantation. As the two methods are expensive, a limited number of patients were chosen for combined PGT and ERA in the study, and observed the improved outcomes in patients who opted for both ERA and PGT. Rubio C and his colleagues conducted a randomized controlled trial with a prospective study design and reported the importance of preimplantation genetic screening (PGS) in RIF patients with a success rate of 47.9% vs. 27.9% OPRs (P=0.0402) and 36.6% vs. 22.1% IRs (P=0.0112) in PGS group versus unscreened blastocyst group. So, their study results represented that embryonic abnormalities would account for 20% failure of PR and 14% ineffective IR. The current study is the first study from India demonstrating the significant role of PGT compared to ERA in RIF patients.

The study requires PGT or ERA in repeated implantation failure patients of the IVF, as the body makeup of the Indian population is not suitable for perfect pregnancy outcomes due to their late marriage, increased pollution, and improper nutrition in rural people. The following were the reasons for including a limited number in the control group compared to the study case group:

The first reason was- the participants were not interested to go for PGT and ERA, as the methods are not affordable based on their financial status and even the non-availability of medical insurance policies for IVF clinical procedures. However, we provided proper counseling and guidance to participants later on regarding the importance of IVF, ERA, and PGT in improving the pregnancy outcomes leading to an increase in the number of patients in IVF. Therefore, more numbers participated in group II (case group - 219).

The second reason was- many of the patients were not willing to participate in the control group. Therefore, very less a number in group I (control arm-72). As the available tests like ERA and PGT analysis give better pregnancy outcomes in IVF, many patients opted for either or both the tests, irrespective of expenses, after we counseled the patients. 

The third reason was- the study participants did their previous IVF treatments in other centres, and we had limited data on past cycles of treatment. Since our IVF clinic is a tertiary care centre, patients referred here from other centres directly opt for both PGT or ERA rather than IVF alone as there is an unavailability of PGT or ERA tests in those centres. This might be the cause of poor recruitment of patients in the control arm.

This study also determined the significant role of gamete types in implantation, as high IRs were noted in the group of subjects who opted for donor oocytes. The primary reasons for failed implantation in ART cycles are genetic abnormalities and aneuploidy, so PGT-A is recommended for all RIF patients. While we recommended PGT-A for all subjects, only some of them consented to aneuploidy screening. There is remarkable implantation and CPR in patients who opted for PGT-A screening, irrespective of their ERA outcome, compared to those who did not receive PGT-A screening. In addition, the embryos screened by PGT-A in the receptive endometrial group resulted in high implantation compared to morphologically screened embryos. This clearly shows that, in RIF and endometrial factors, knowing the embryo's ploidy status is a major factor in achieving implantation in this group of patients. However, the present study included a smaller number of participants who opted for PGT-A, and to confirm the significance of PGT-A in RIF patients, it needs a larger sample size to validate the observations.

The study reported a high AR in the nonreceptive group, and we further analyzed data to assess whether the patients who experienced abortion opted for PGT-A. Interestingly, all the embryos in the abortion group under the nonreceptive category were screened using embryo morphology, and none opted for PGT-A screening to select embryos for transfer. This further supports the importance of embryo screening by PGT-A in RIF patients.

We anticipated that asynchrony between the euploid embryo and uterine receptivity might be the primary cause of the failure. Implantation is a complex mechanism of immunological, anatomical, endometrial, embryogenic, and many more unknown factors. For patients with implantation failure, despite good endometrial receptivity, euploid embryos, and no other known contributing factors, assessment is challenging and should be studied further to identify the causes.

The study determined the importance of PGT-A and ERA, which offer significant benefits in clinical outcomes during the cycle with positive pregnancy rates. Testing the embryo's genetic quality and endometrium receptivity significantly contributed to successful implantation.

Our study had several important limitations. The study design was retrospective and conducted in a single center. The sample size is small, making interpretations from the ERA nonreceptive group inconclusive compared to the ERA receptive group. Larger prospective studies would offer more statistical power to support our findings. As a small number of patients opted for PGT-A along with ERA testing in the present study, it is recommended for all participants to do both PGT-A and ERA in future prospective studies.

## Conclusions

The data set from the present study reveals the significant role of ERA along with PGT in achieving better clinical outcomes for the first time in Indian patients. An ERA offered a personalized approach to identifying the WOI, adjusting the progesterone exposure, and then performing embryo transfer accordingly. There is an improvement in IR and CPR in RIF patients with a displaced WOI by transferring euploid embryos in a PET cycle. An ERA is a promising technique for RIF patients even after euploid embryo transfer. Further, PGT-A benefits RIF significantly, particularly in cases with optimal endometrial receptivity.
